# Association between *CD209 -336A/G* and *-871A/G* Polymorphisms and Susceptibility of Tuberculosis: A Meta-Analysis

**DOI:** 10.1371/journal.pone.0041519

**Published:** 2012-07-24

**Authors:** Kai Chang, Shaoli Deng, Weiping Lu, Feng Wang, Shuangrong Jia, Fake Li, Lili Yu, Ming Chen

**Affiliations:** 1 Department of Laboratory Medicine, Institute of Surgery Research, Daping Hospital, Third Military Medical University, Chongqing, China; 2 Department of Obstetrics and Gynecology, Institute of Surgery Research, Daping Hospital, Third Military Medical University, Chongqing, China; St. Petersburg Pasteur Institute, Russian Federation

## Abstract

**Background:**

The association between *CD209* promoter polymorphisms (*-336A/G, -871A/G*) and tuberculosis (TB) risk has been widely reported, but results of previous studies remain controversial and ambiguous. To assess the association between *CD209* polymorphisms and TB risk, a meta-analysis was performed.

**Methods:**

Based on comprehensive searches of the PubMed, Embase, Web of Science, Weipu, and CBM databases, we identified outcome data from all articles estimating the association between *CD209* polymorphisms and TB risk. The pooled odds ratio (OR) with 95% confidence intervals (CIs) were calculated.

**Results:**

A total of 14 studies with 3,610 cases and 3,539 controls were identified. There was no significant association between *CD209 -336A/G* polymorphism and TB risk (OR = 1.04, 95% CI = 0.91–1.19 for G vs. A; OR = 1.13, 95% CI = 0.84–1.53 for GG vs. AA; OR = 1.04, 95% CI = 0.87–1.24 for GG+AG vs. AA; OR = 1.11, 95% CI = 0.88–1.39 for GG vs. AG+AA). However, the significant association was revealed for Asians in GG vs. AA (OR = 2.48, 95% CI = 1.46–4.22, P = 0.0008) and GG vs. AG+AA (OR = 2.10, 95% CI = 1.33–3.32, P = 0.001). For the *CD209 -871A/G* polymorphism, lack of an association was also found (OR = 0.81, 95% CI = 0.70–0.95 for G vs. A; OR = 1.00, 95% CI = 0.52–1.93 for GG vs. AA; OR = 0.73, 95% CI = 0.60–0.89 for GG+AG vs. AA; OR = 1.09, 95% CI = 0.57–2.10 for GG vs. AG+AA).

**Conclusion:**

The present meta-analysis suggested that *CD209* promoter polymorphisms (*-336A/G, -871A/G*) were unlikely to substantially contribute to TB susceptibility. However, the *GG* genotype of *CD209 -336A/G* polymorphism might be a genetic risk factor that increases TB susceptibility for Asians in GG vs. AA and GG vs. AG+AA.

## Introduction

Tuberculosis (TB) constitutes a serious threat to public health throughout the world, particularly in developing countries [1]. Recent data from the World Health Organization (WHO) show that nearly 10 million new cases arise and 1.7 million deaths die of TB annually [2]. It has been reported that one-third of population is infected with *Mycobacterium* tuberculosis; however, only one-tenth of infected individuals will ever develop active TB [3]. Thus, host genetic susceptibility, combined with environmental factors, may play a crucial role in exploring the infection mechanism of *Mycobacterium* tuberculosis [4]–[5].

The *CD209* gene, located on human chromosome 19p13.2–3, is composed of 7 exons and 6 introns, and is about 13 kb in length [6]–[7]. This gene encoded Dendritic Cell-Specific ICAM3-Grabbing Non-integrin (DC-SIGN), which is one of the major receptor of *Mycobacterium* tuberculosis on human dendritic cells [8]. In addition, the *Mycobacterium* tuberculosis interacts with DC-SIGN to activate the Raf-1-acetylation-dependent signaling pathway that is involved in the regulation of adaptive immune response to tuberculosis. Furthermore, the DC-SIGN could suppress Toll-like receptor signaling leading to cytokine secretion. This effect may be a part of immune evasion to TB progression [9]–[10]. Therefore, the *CD209* gene might play a crucial role in host immunity to TB and might be one of the candidate genes for susceptibility of TB.

A relatively large number of studies evaluated the association between *CD209* polymorphisms (*-336A/G, -871A/G*) and TB risk, but the results have been inconsistent due to limited sample sizes and different study populations. To derive a more comprehensive and precise estimation of the relationship, we carried out a meta-analysis on all eligible case-control studies to estimate the effect of *CD209* polymorphisms on the risk of TB and to quantify the potential between-study heterogeneity.

## Results

### Study Characteristics

Eleven publications, including 3,610 cases and 3,539 controls, met the inclusion criteria [6], [11]–[20]. A flowchart detailing the process for study identification and selection was shown in Fig. S1. The publication of Vannberg et al. presented four independent case-control studies, each study was considered separately for analysis. Therefore, 11 publications including 14 studies were involved in this meta-analysis. The main characteristics of the studies were shown in Table 1. The sample sizes ranged from 273 to 1093 patients (median 390, IQR 324–669). Nine of the 14 included studies reported the proportion of male patients, which ranged from 14.5% to 81.1% (median 62.25%, IQR 53.225%–68.5%). Thirteen of the 14 included studies clearly described the diagnostic criteria. One study didn’t provide the genotype number [18]. Gene frequencies of all the individual studies were shown in Table S1. The NOS scores ranged from 7 to 9, which indicated that the methodological quality was generally good. The quality assessment of included studies was shown in Table S2. The genotype distribution in the controls of all studies was in agreement with HWE.

**Table 1:** Association between individual study characteristics and *CD209* gene polymorphisms.

Study	Origin	Ethnicity	Male patients (%)	Mean age(years)		Sample types	Sample size		Polymorphisms investigated	Clinical diagnoses performed	Control source	Genotypingmethod	Score
				Cases	Controls		Cases	Controls					
Kobayashi et al. [12]	Indonesian	Asian	53.7	41.6±15.4	39.3±12.7	PTB	532	561	-336A/G, -871A/G	Smear, radiologic,clinical symptoms	Healthy individuals	Sequencing	8
Ogarkov et al. [17]	Russian	Caucasian	76.3	42.3±12.1	41.9±9.2	PTB	101	177	-336A/G	NR	Healthy individuals	Taq Man LNA technology	7
						EPTB	90						
Zheng et al. [19]	Chinese	Asian	65.4	44.6±17.7	NR	PTB	237	244	-336A/G, -871A/G	Culture, radiologic	Healthy individuals	Sequencing	7
Sadki et al. [15]	Moroccan	Mixed	81.1	33.7±13.2	NR	PTB	122	151	-336A/G	Smear, culture,histology, radiologic,clinical symptoms	Healthy unrelateddonors	Taq Man SNP genotyping assays	8
Selvaraj et al. [13]	Indian	Caucasian	61.2	34.0±8.2	30.6±8.3	PTB	183	157	-336A/G	Smear, culture,radiologic, clinicalsymptoms	Healthy individuals	PCR-RFLP	7
						EPTB	31						
Zhuang et al. [20]	Chinese	Asian	65.9	43(16–77)	30(15–78)	PTB	167	167	-336A/G	Smear, culture,histology, radiologic,clinical symptoms	Healthy unrelateddonors with nohistory ofautoimmunedisease	SSP-PCR	7
Vannberg et al. (a) [14]	Gambian	African	NR	NR	NR	PTB	676	327	-336A/G	Smear, culture,histology	Healthy unrelateddonors	MALDI-TOF	8
Vannberg et al. (b) [14]	Guinean	African	NR	NR	NR	PTB	151	180	-336A/G	Smear, culture,histology	Healthy unrelated donors	MALDI-TOF	8
Vannberg et al. (c) [14]	Guinea-Bissau	African	NR	NR	NR	PTB	162	141	-336A/G	Smear, culture,histologyconfirmed TB	Healthy unrelateddonors	MALDI-TOF	8
Vannberg et al. (d) [14]	Malawian	African	NR	NR	NR	PTB	244	295	-336A/G	Smear, culture,histologyconfirmed TB	Healthy unrelated donors	MALDI-TOF	8
Ben-Ali et al. [18]	Tunisian	Mixed	NR	NR(18–65)	NR(25–60)	NR	138	140	-336A/G, -871A/G	Smear, culture,radiologic, clinicalsymptoms confirmedTB	Healthy unrelateddonors	Sequencing	8
Olesen et al. [16]	Guinea-Bissau	African	60.4	37.3	38.1	PTB	315	340	-336A/G	Smear, culture,histology, radiologic,clinical symptomsconfirmed TB	Healthy unrelateddonors	Taq Man SNP genotyping assays	9
Barreiro et al. [6]	SouthAfrican	African	51.8	36.7±10.9	34.6±12.5	PTB	351	360	-336A/G, -871A/G	Smear, cultureconfirmed TB	Healthy unrelateddonors	Taq Man or fluorescence polarization	8
Gómez et al. [11]	Colombian	Mixed	14.5	40.0±15.0	NR	NR	110	299	-336A/G	Smear, cultureconfirmed TB	Healthy unrelateddonors with nohistory ofautoimmunedisease	Sequencing	8

Abbreviations and definitions: PTB, pulmonary tuberculosis; EPTB, extra-pulmonary tuberculosis; NR, not report; PCR-RFLP, Polymerase Chain Reaction-Restriction Fragment Length Polymorphism; SSP-PCR, sequence specific primer-Polymerase Chain Reaction; MALDI-TOF, Matrix-Assisted Laser Desorption/Ionization Time of Flight Mass Spectrometry.

### The CD209 *-336A/G* Alleles and Tuberculosis Susceptibility

**Figure 1:**
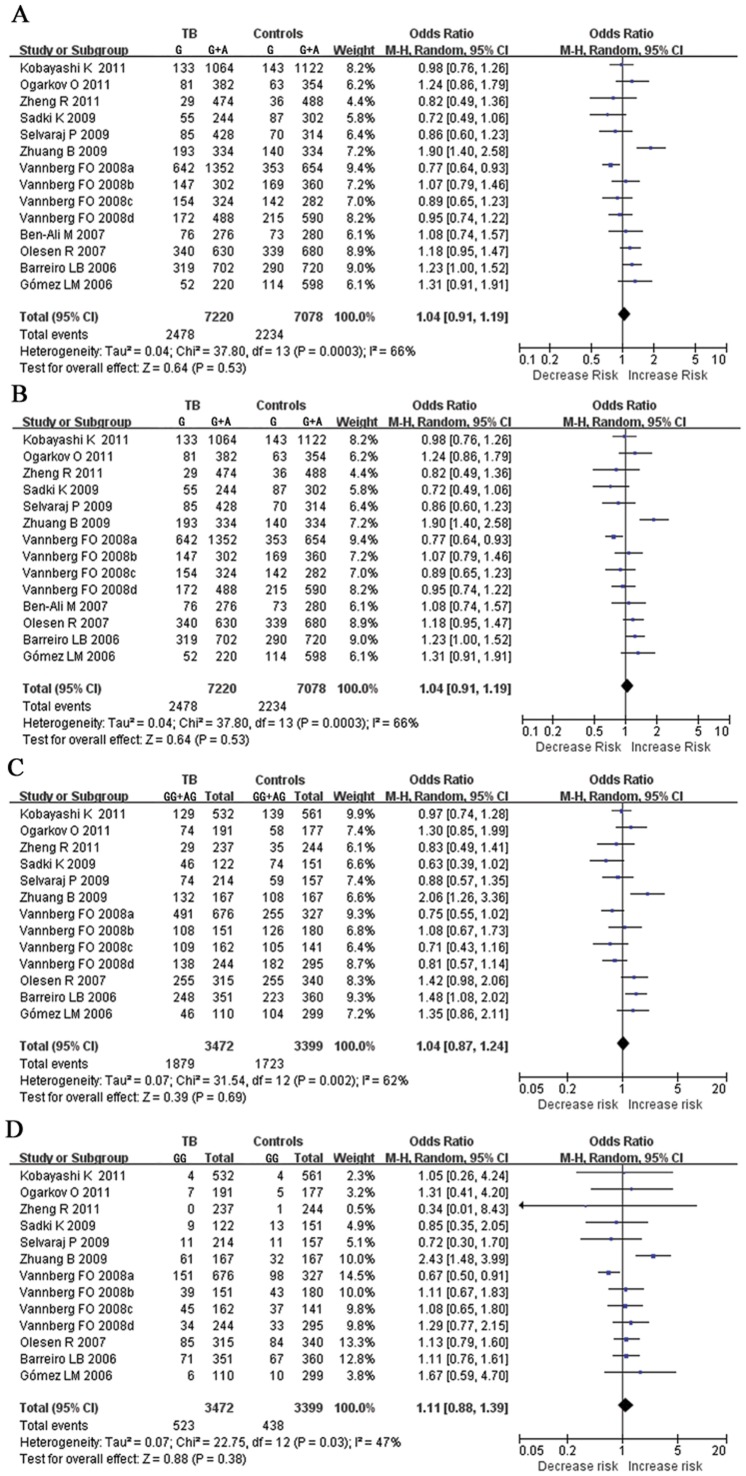
Forest plot of the overall risk of TB associated with the *CD209 -336A/G* promoter polymorphism. No significant association was found between the *CD209* -336A/G polymorphism and TB risk in all genetic models. A, G vs. A; B, GG vs. AA; C, dominant genetic model; D, recessive genetic model. Error bars indicate 95% CI. Solid squares represent each study in the meta-analysis. Solid diamonds represent pooled OR.

Random effects models were used to calculate the pooled OR in all genetic models. Overall, the combined results showed that no significant association was found in all genetic models (OR = 1.04, 95% CI = 0.91–1.19 for G vs. A, OR = 1.13, 95% CI = 0.84–1.53 for GG vs. AA, OR = 1.04, 95% CI = 0.87–1.24 for GG+AG vs. AA, and OR = 1.11, 95% CI = 0.88–1.39 for GG vs. AG+AA). Forest plots on the basis of all studies were shown in Fig. 1A, B, C, D. When stratified by ethnicity, we observed a wide variation of G allele frequencies between the controls across different ethnicities. The G allele frequencies were significant difference in Africans, Asians, Caucasians and Mixed populations (P = 0.007). Forest plots were shown in Fig. S2. When meta-analysis was performed to assess association between *CD209 -336A/G* polymorphism and TB risk in different ethnicities, significant association was revealed for Asians in GG vs. AA (OR = 2.48, 95% CI = 1.46–4.22, P = 0.0008) and GG vs. AG+AA (OR = 2.10, 95% CI = 1.33–3.32, P = 0.001) (Fig. S3, Fig. S4, Fig. S5, and Fig. S6). On subgroup analysis by sample types, no evidence of association was found in all genetic models for pulmonary tuberculosis (PTB) and extra-pulmonary tuberculosis (EPTB). The results were shown in Table 2.

**Table 2:** Meta-analyses of *CD209 -336A/G* promoter polymorphism and risk of TB in each subgroup.

Category	G vs. A	GG vs.AA	Dominant model	Recessive model
	OR (95%CI) *I* ^2^ (%)	OR (95%CI) *I* ^2^ (%)	OR (95%CI) *I* ^2^ (%)	OR (95%CI) *I* ^2^ (%)
Ethnicity				
African	1.00 (0.85–1.19) 65	1.03 (0.75–1.42) 61	1.00 (0.76–1.32) 69	0.97 (0.83–1.15) 42
Asian	1.17 (0.71–1.94) 85	2.48 (1.46–4.22) 46	1.17 (0.71–1.93) 75	2.10 (1.33–3.32) 20
Caucasian	1.03 (0.80–1.33) 49	0.91 (0.45–1.82) 0	1.07 (0.79–1.45) 37	0.89 (0.45–1.78) 0
Mixed	1.01 (0.72–1.43) 60	1.03 (0.52–2.05) 45	0.93 (0.44–1.95) 80	1.11 (0.56–2.18) 0
Sample types				
PTB	1.02 (0.88–1.18) 69	1.08 (0.79–1.48) 62	1.01 (0.83–1.22) 62	1.08 (0.85–1.37) 51
EPTB	1.21 (0.84–1.74) 0	1.51 (0.58–3.92) 0	1.20 (0.77–1.86) 30	1.51 (0.59–3.89) 0

Abbreviations and definitions: PTB, pulmonary tuberculosis; EPTB, extra-pulmonary tuberculosis; CI, 95% confidence intervals; OR, odds ratio.

### The CD209 *-871A/G* Alleles and Tuberculosis Susceptibility

**Figure 2:**
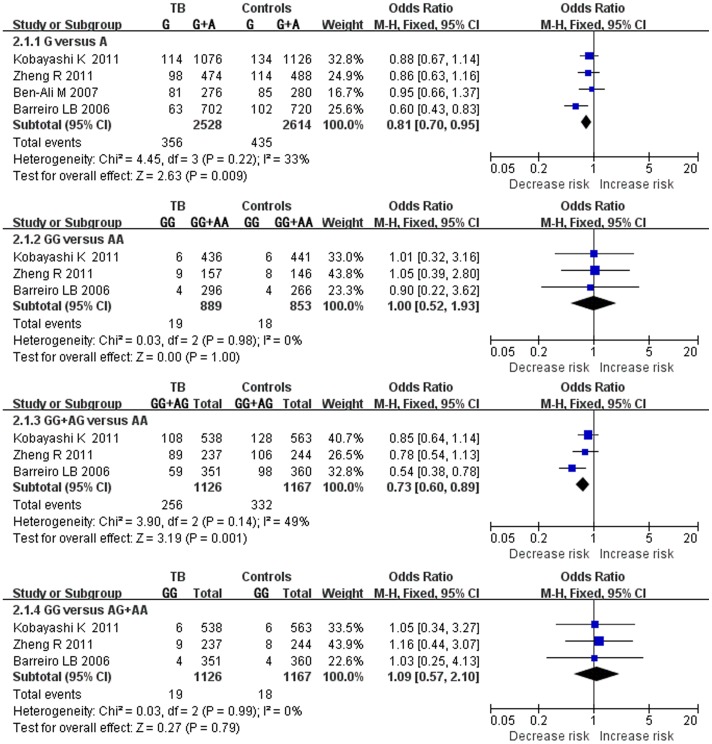
Forest plot of the overall risk of TB associated with the *CD209 -871A/G* promoter polymorphism. No significant association was found between the *CD209 -871A/G* polymorphism and TB risk in all genetic models. Error bars indicate 95% CI. Solid squares represent each study in the meta-analysis. Solid diamonds represent pooled OR.

The results on the *CD209 -871A/G* polymorphism indicated that the G allele had no significant association to TB susceptibility as compared to the A allele under the fixed effects models (Fig. 2). The results were as followed: G vs. A (OR = 0.81, 95% CI = 0.70–0.95), GG vs. AA (OR = 1.00, 95% CI = 0.52–1.93), GG+AG vs. AA (OR = 0.73, 95% CI = 0.60–0.89), GG vs. AG+AA (OR = 1.09, 95% CI = 0.57–2.10).

### Heterogeneity Analysis

**Figure 3:**
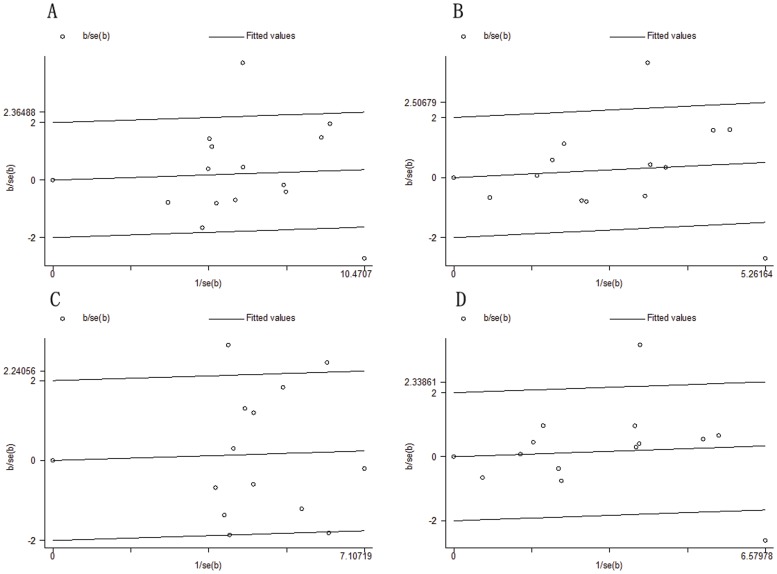
Galbraith plot of *CD209 -336A/G* promoter polymorphism and TB risk. A, The two studies of Zhuang et al. and Vannberg FO(a) et al. were outliers in G vs. A; B, The two studies of Zhuang et al. and Vannberg FO(a) et al. were outliers in GG vs. AA; C, The three studies of Zhuang et al., Vannberg FO(a) et al. and Barreiro LB et al. were outliers in dominant genetic model; D, The two studies of Zhuang et al. and Vannberg FO(a) et al. were outliers in recessive genetic model.

For *CD209 -336A/G* polymorphism, there were statistically significant heterogeneity in G vs. A (*I* ^2^ = 66%, *P*_heterogeneity_ = 0.0003), GG vs. AA (*I* ^2^ = 60%, *P*_heterogeneity_ = 0.003), dominant genetic model (*I* ^2^ = 62%, *P*_heterogeneity_ = 0.002), and recessive genetic model (*I* ^2^ = 47%, *P*_heterogeneity_ = 0.03). To explain the heterogeneity, Galbraith plots were performed in all genetic models. The two studies of Zhuang et al. and Vannberg FO(a) et al. were outliers in the G vs. A, GG vs. AA, and recessive genetic model (Fig. 3A, B, D). The three studies of Zhuang et al., Vannberg FO(a) et al. and Barreiro LB et al. were outliers in the dominant genetic model (Fig. 3C). When the studies of Zhuang et al., Vannberg FO(a) et al. and Barreiro LB et al. were excluded respectively, all *I* ^2^ values were less than 50% and *P*_heterogeneity_ were greater than 0.1 (Table 3). The significant of pooled OR in all genetic models was not influenced after excluding the studies. By meta-regression analysis, the heterogeneity sources were attributed to the sample size, control source, NOS scores, and the frequency of G allele. However, the four above-mentioned factors had no significant impact on pooled OR in all genetic models (Table S3).

**Table 3:** Meta-analyses of *CD209 -336A/G* promoter polymorphism and risk of TB after omitting the studies.

Polymorphism	OR(95% CI)	Z	P _OR_	*I* ^2^ (%)	*P*_heterogeneity_	Effectmodel
G vs. A^a^	1.05(0.96, 1.14)	1.12	0.26	17	0.28	F
GG vs. AA^a^	1.16(0.95, 1.42)	1.46	0.15	0	0.74	F
GG+AG vs. AA^b^	0.98(0.86, 1.11)	0.29	0.77	36	0.12	F
GG vs. AG+AA^a^	1.11(0.93, 1.32)	1.14	0.26	0	0.98	F

Abbreviations and definitions: TB, tuberculosis; CI, 95% confidence intervals; OR, odds ratio; *P*_heterogeneity,_ P value of Q test for heterogeneity; F, fixed-effect models.

a *CD209 -336A/G* promoter polymorphism and risk of TB after excluding the two studies of Zhuang et al. and Vannberg FO(a) et al.

b *CD209 -336A/G* promoter polymorphism and risk of TB after excluding the three studies of Zhuang et al., Vannberg FO(a) et al. and Barreiro LB et al.

### Sensitivity Analysis

Sensitivity analysis was performed though sequential excluding individual studies. Statistically similar results were obtained in all genetic models after sequentially excluding each study, indicated that our dates were stability and liability in this meta-analysis.

### Publication Bias

Publication bias was estimated by the funnel plots. As shown in Fig. S7, the shape of the funnel plots revealed asymmetry in some degree due to the limited number of literatures. Then, Egger’s linear regression test was used to provide statistical evidence of funnel plots asymmetry. For *CD209 -336A/G* polymorphism, the P values of Egger’s linear regression test were 0.789, 0.880, 0.951, and 0.606 for G vs. A, GG vs. AA, dominant genetic model, and recessive genetic model, respectively. For *CD209 -871A/G* polymorphism, the P values were 0.779, 0.117, 0.516, and 0.302, respectively. The result still did not suggest any evidence of publication bias.

## Discussion

According to WHO, The estimates of TB global burden were 9.4 million incident cases, 14 million prevalent cases, and 1.68 million deaths (with 0.38 million in HIV-positive individuals) in 2009 [21]. So, it is necessary to explore the infection mechanism. Clearly, the genetic contribution of the host plays a significant role in determining susceptibility to TB [22]. The *CD209* gene, encoding DC-SIGN, might play a crucial role in the pathogenesis of TB. The association between *CD209* gene polymorphisms and TB risk was first reported in a South African populations by Barreiro et al [6]; however, as discussed above, conflicting data regarding the role of *CD209* in TB susceptibility and presentation have been reported [12]–[15], [19]. Against this backdrop, we performed a meta-analysis to clarify the relationship between *CD209* polymorphisms and TB risk.

In this meta-analysis, 14 studies (14 subgroups for *-336A/G* polymorphism, 4 subgroups for *-871A/G* polymorphism) on *CD209* gene were performed to provide the most comprehensive assessment of the relationship between polymorphisms and TB. For *CD209 -336A/G* polymorphism, the G allele of *-336A/G* polymorphism had no association with the TB susceptibility for the G vs. A, GG vs. AA, dominant genetic model, and recessive genetic model in overall populations. For the *CD209 -871A/G* polymorphism, lack of an association was also found. Currently, many meta-analyses have reported the association between gene polymorphisms and TB susceptibility. Compared with the *CD209* gene polymorphisms, the interferon-γ (*IFN-γ*), natural resistance-associated macrophage protein 1(*NRAMP1*), and Vitamin D receptor (*VDR*) gene polymorphisms contribute to susceptibility to TB [23]–[25]. The *P2X7,* Mannose-binding lectin 2 (*MBL2*), tumor necrosis factor-α (*TNF-α*), and Interleukin-10 (*IL-10*) gene polymorphisms are not associated with TB risk [26]–[29].

In view of the complex effect of genetic polymorphisms on disease progression, the lack of an association between *CD209* polymorphisms and TB susceptibility may attribute to other polymorphisms in *CD209* gene promoter which could affect the expression of DC-SIGN. In current study, we also performed meta-analysis to identify the association between *CD209 -939G/A* polymorphism and TB risk. There was no association between *CD209 -939G/A* polymorphism and TB risk (Fig. S8). de Wit E et al. [30] found that two interactions (*DC-SIGN -871 A/G*: *NRAMP1(GT)_n_* repeat and *DC-SIGN -871 A/G:MBL*) influenced the risk of developing TB. Thus, the interaction between gene and gene might play a crucial role in the association of *CD209* polymorphisms with TB risk.

Due to different racial or ethnic populations with different frequencies of alleles, different genetic backgrounds may affect TB susceptibilities. Therefore, subgroup analyses were performed according to ethnicity. First, we detected whether there was G allele frequency of variation in different ethnicities. For *CD209 -336A/G* polymorphism, the G allele frequency has significant differences in different populations. Next, the association between *CD209 -336A/G* polymorphism and different ethnicities was explored. The significant association was revealed for Asians in GG vs. AA (OR = 2.48, 95% CI = 1.46–4.22, P = 0.0008) and GG vs. AG+AA (OR = 2.10, 95% CI = 1.33–3.32, P = 0.001). The ethic-dependent association may attribute to interplay among different human alleles, locally predominant, and endemic mycobacterial lineages [31]. Ogarkov et al. [17] demonstrated that the G allele of *CD209 -336A/G* polymorphism could increase the risk of infection with Mycobacterium tuberculosis Beijing but not non-Beijing strain in Russian male population. Thus, the TB in different host infected with the same genotype may manifest as different outcome in clinic.

In our meta-analysis, obvious heterogeneity was observed for *CD209 -336A/G* polymorphism. Then, we used the Galbraith plots to explore the sources of heterogeneity. We found all of the *I* ^2^ values were less than 50% and *P*_heterogeneity_ were greater than 0.1 after excluding the studies of Zhuang et al., Vannberg FO(a) et al. and Barreiro LB et al, respectively. The results indicated that the three studies might be the major source of the heterogeneity for the *CD209 -336A/G* polymorphism. The results of subgroup analyses revealed that the ethnicity and sample type might contribute to the potential heterogeneity. Owing to the limited number of studies in this meta-analysis, we restricted meta-regression analysis to four factors (sample size, control source, NOS scores, and the frequency of G allele), which are the most likely to cause the heterogeneity between studies. However, the four above-mentioned factors had no significant impact on the heterogeneity.

There are some limitations to this meta-analysis. Firstly, the retrieved literature is potentially not comprehensive enough. We did not track the unpublished articles to obtain data for analysis. The potential effect of this publication bias is unknown. Secondly, the small sample sizes in some subgroup analyses may have limited statistical power to estimate the possible risk for *CD209* polymorphisms. Thirdly, TB is a multifactorial disease and potential interactions among gene-gene and gene-environment should be considered. Moreover, as many other factors such as age or gender may participate in the progression of TB, we did not carry out subgroup analysis based on these factors due to limited data.

Conclusively, the *CD209* promoter polymorphisms (*-336A/G, -871A/G*) may lack association with genetic susceptibility of TB. However, genotype *GG* of *CD209 -336A/G* polymorphism might play a role as risk factor for Asians in GG vs. AA and GG vs. AG+AA, which indicated the different genetic backgrounds may affect TB susceptibilities. Moreover, further studies with large sample size of different ethnic populations will be necessary to combine genetic factors together with poor economic conditions, malnutrition, stress and overcrowding.

## Materials and Methods

### Data Sources and Search Strategy

This meta-analysis followed the Preferred Reporting Items for Systematic Reviews and Meta-analysis (PRISMA) criteria [32]. Two investigators (K.C. and S.D.) independently performed a systematic electronic search of the PubMed, Embase, Web of Science, Weipu, and CBM databases for original articles published until 1 December, 2011 to identify potentially relevant articles and abstracts. Search terms used were “*CD209* or DC-SIGN” and “tuberculosis or TB” and “polymorphism or mutation or variant”. There were no language restrictions. We reviewed the bibliographies of all selection articles to identify additional relevant studies.

### Selection of Publications

Two reviewers (K.C. and W.L.) independently screened titles and abstracts of all studies for relevancy. Disagreements were resolved by a third opinion (M.C.). Full-text publications were retrieved for relevant articles. The strength of the individual studies was weighed for relevance, based on the following items: (1) evaluation of the *CD209 -336A/G* or *-871A/G* polymorphisms and TB risk, (2) case-control studied (family-based study design with linkage considerations was excluded), (2) sufficient data for estimating an odds ratio (OR) with 95% confidence intervals (CIs), (3) genotype distribution of control population in Hardy-Weinberg equilibrium (HWE), and (4) studies written in English or Chinese. For the studies with the same or overlapping data by the same authors, the most recent or largest population was selected.

### Data Extraction

Data were extracted independently from each study by two reviewers (K.C. and S.D.) according to the inclusion criteria listed above. Agreement was reached after discussion for conflicting data. The following data were collected from each study: first author’s name, publication year, original country, ethnicity, sample size, sample types, TB definition, genotyping method, and genotype number in cases and controls.

### Quality Assessment

The quality of included studies was assessed independently by the same two investigators using the Newcastle-Ottawa Scale (NOS) (Stang A., 2010). The NOS uses a ‘star’ rating system to judge quality based on 3 aspects of the study: selection, comparability, and exposure. Scores were ranged from 0 stars (worst) to 9 stars (best). Studies with a score of 7 stars or greater were considered to be of high quality. Disagreement was settled as described above.

### Statistical Analysis

The strength of association between *CD209* polymorphisms and TB risk was estimated by OR and corresponding 95% CIs. The pooled OR was calculated respectively for G vs. A, GG vs. AA, dominant genetic model (GG+AG vs. AA), and recessive genetic model (GG vs. AG+AA). Between-study heterogeneity was assessed by the Q-test and *I* ^2^ test, *P*_heterogeneity_<0.10 and *I* ^2^>50% indicated evidence of heterogeneity. Then, the random-effects model (the DerSimonian and Laird method) [33]–[34] was used to calculate the pooled OR. Otherwise, the fixed-effects model (Mantel-Haenszel) [35] was adopted. Subgroup analyses and meta-regression were used to analyze the sources of heterogeneity.

Sensitivity analysis was mainly performed to assess the stability of the results, namely, a single study in the meta-analysis was deleted each time to reflect the influence of the individual data set to the pooled OR. Asymmetry funnel plots were inspected to assess potential publication bias. The Egger’s linear regression test [36] was also used to assess publication bias statistically.

Data were analyzed by using STATA 11.0 (Stata Corporation, College Station, TX, USA) and Revman 5.0 (The Cochrane Collaboration).

## Supporting Information

Figure S1 **Flow diagram of the selection of eligible studies.**
(TIF)

Figure S2 **Frequencies of the minor allele (G allele) of the** ***CD209 -336A/G*** **polymorphism among controls subjects stratified by ethnicity.** The G allele frequencies were significant difference in Africans, Asians, Caucasians and Mixed populations (P = 0.007)
(TIF)

Figure S3 **Forest plot of** ***CD209*** **-336A/G promoter polymorphism and risk of TB in G vs. A for each subgroup.** No significant association was found between the *CD209* -336A/G polymorphism and TB risk in G vs. A.
(TIF)

Figure S4 **Forest plot of** ***CD209*** **-336A/G promoter polymorphism and risk of TB in GG vs. AA for each subgroup.** The significant association was revealed for Asians in GG vs. AA (OR = 2.48, 95% CI = 1.46–4.22, P = 0.0008).
(TIF)

Figure S5 **Forest plot of** ***CD209*** **-336A/G promoter polymorphism and risk of TB in dominant model for each subgroup.** No significant association was found between the *CD209* -336A/G polymorphism and TB risk in dominant model.
(TIF)

Figure S6 **Forest plot of** ***CD209*** **-336A/G promoter polymorphism and risk of TB in recessive model for each subgroup.** The significant association was revealed for Asians in recessive model (OR = 2.10, 95% CI = 1.33–3.32, P = 0.001).
(TIF)

Figure S7 **Funnel plots of all genetic models in overall studies.** A. G vs. A; B. GG vs. AA; C. dominant model (GG+AG vs. AA); D. recessive model (GG vs. AG+AA). Funnel plots of dominant model seemed asymmetry. Each point represents a separate study for the indicated association.
(TIF)

Figure S8 **Forest plot of the overall risk of TB associated with the** ***CD209*** **-939G/A promoter polymorphism.** No significant association was found between the *CD209* -939G/A polymorphism and TB risk in all genetic models. Error bars indicate 95% CI. Solid squares represent each study in the meta-analysis. Solid diamonds represent pooled OR.
(TIF)

Table S1 **Gene frequencies of all the individual studies used for the meta-analysis.**
(DOC)

Table S2 **Quality assessment of included studies.**
(DOC)

Table S3 **Meta-regression analysis of CD209 -336A/G promoter polymorphism and risk of TB after omitting the studies.**
(DOC)
