# Supplementary figures and images for: Association between CD209 -336A/G and -871A/G Polymorphisms and Susceptibility of Tuberculosis: A Meta-Analysis
Source: PLoS One. 2012 Jul 24;7(7):e41519. doi: 10.1371/journal.pone.0041519 (PMC3404017; doi:10.1371/journal.pone.0041519)

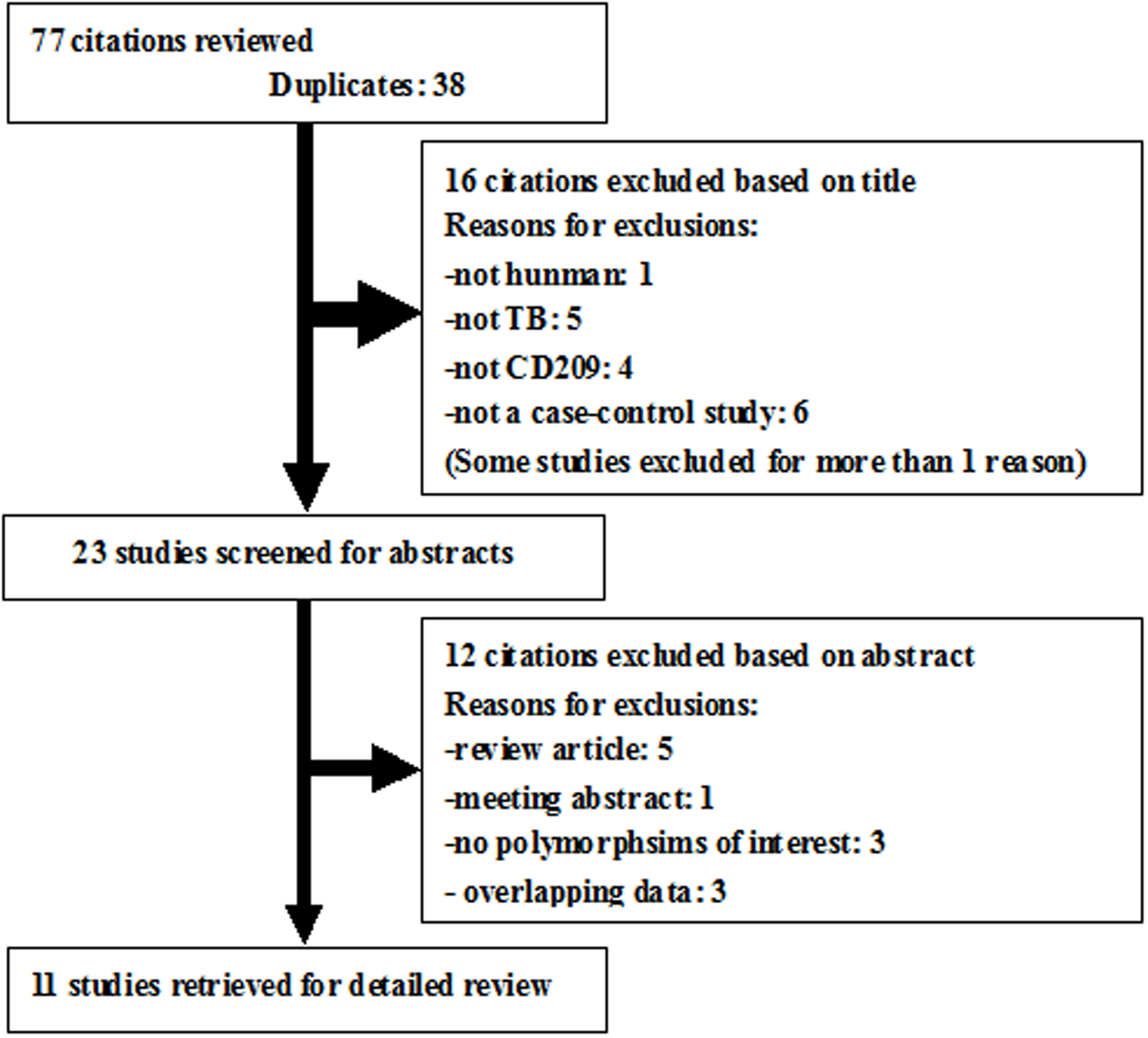

Supplement: Figure S1 — Flow diagram of the selection of eligible studies. (TIF) [file pone.0041519.s001.tif]

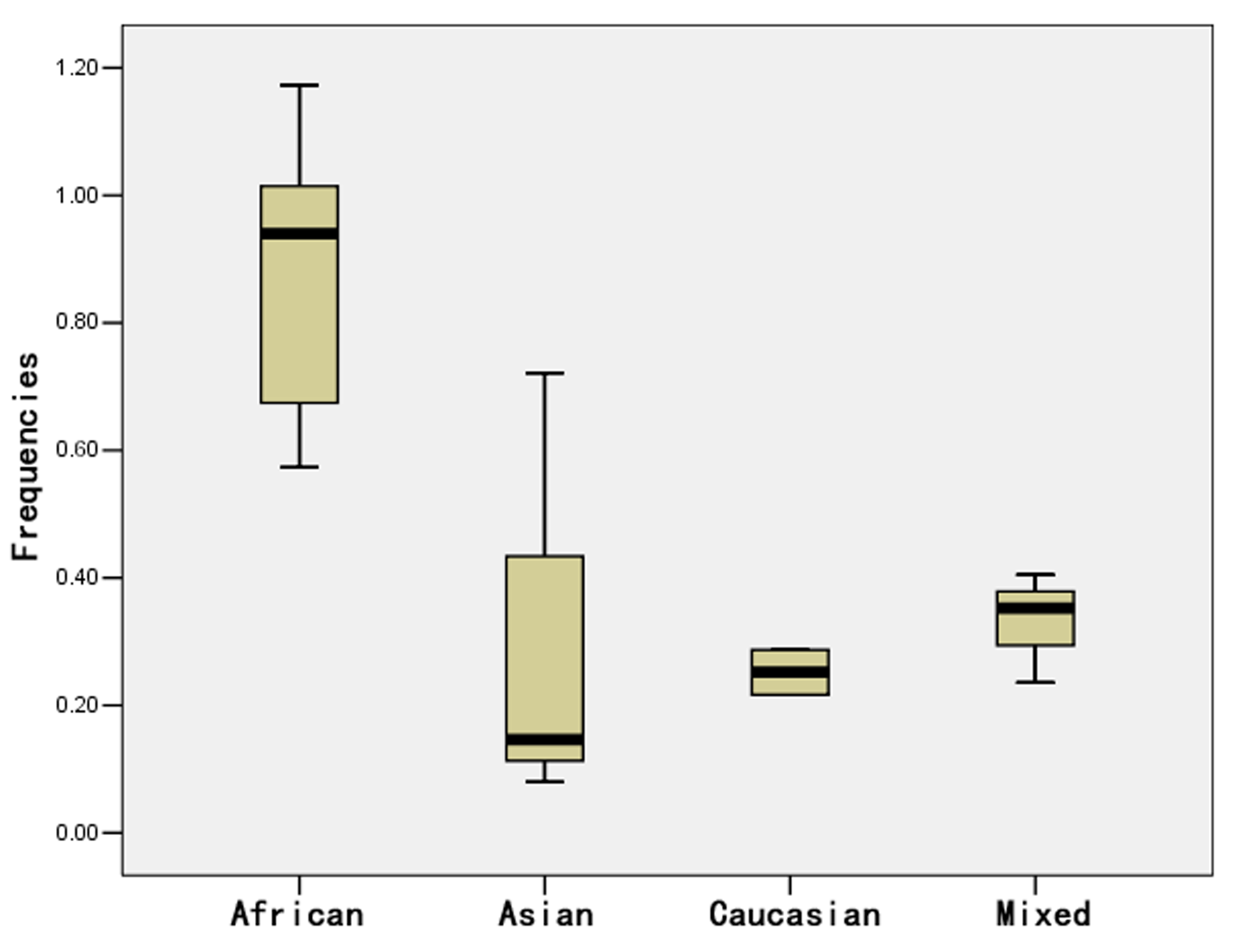

Supplement: Figure S2 — Frequencies of the minor allele (G allele) of the CD209 -336A/G polymorphism among controls subjects stratified by ethnicity. The G allele frequencies were significant difference in Africans, Asians, Caucasians and Mixed populations (P = 0.007) (TIF) [file pone.0041519.s002.tif]

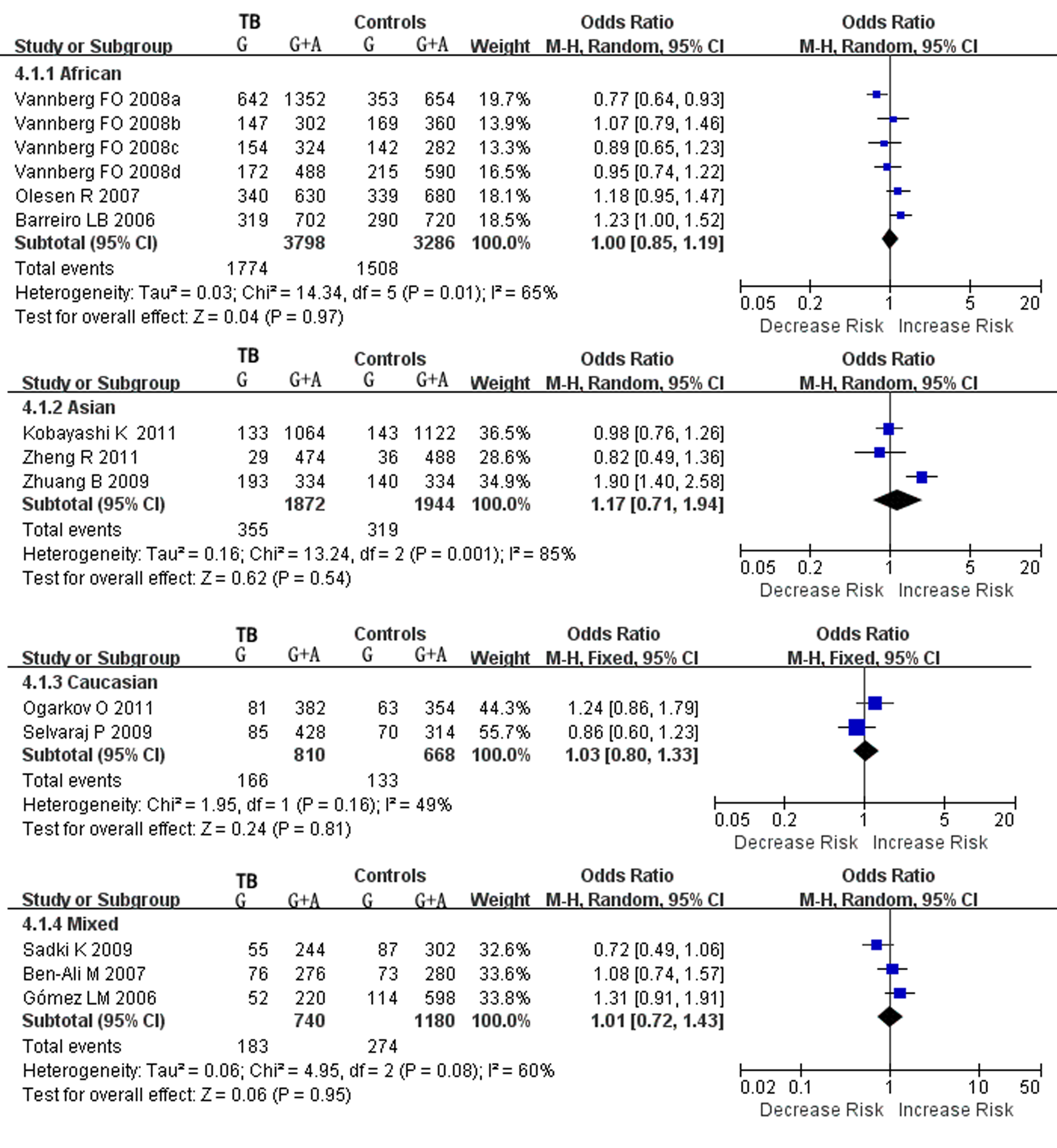

Supplement: Figure S3 — Forest plot of CD209 -336A/G promoter polymorphism and risk of TB in G vs. A for each subgroup. No significant association was found between the CD209 -336A/G polymorphism and TB risk in G vs. A. (TIF) [file pone.0041519.s003.tif]

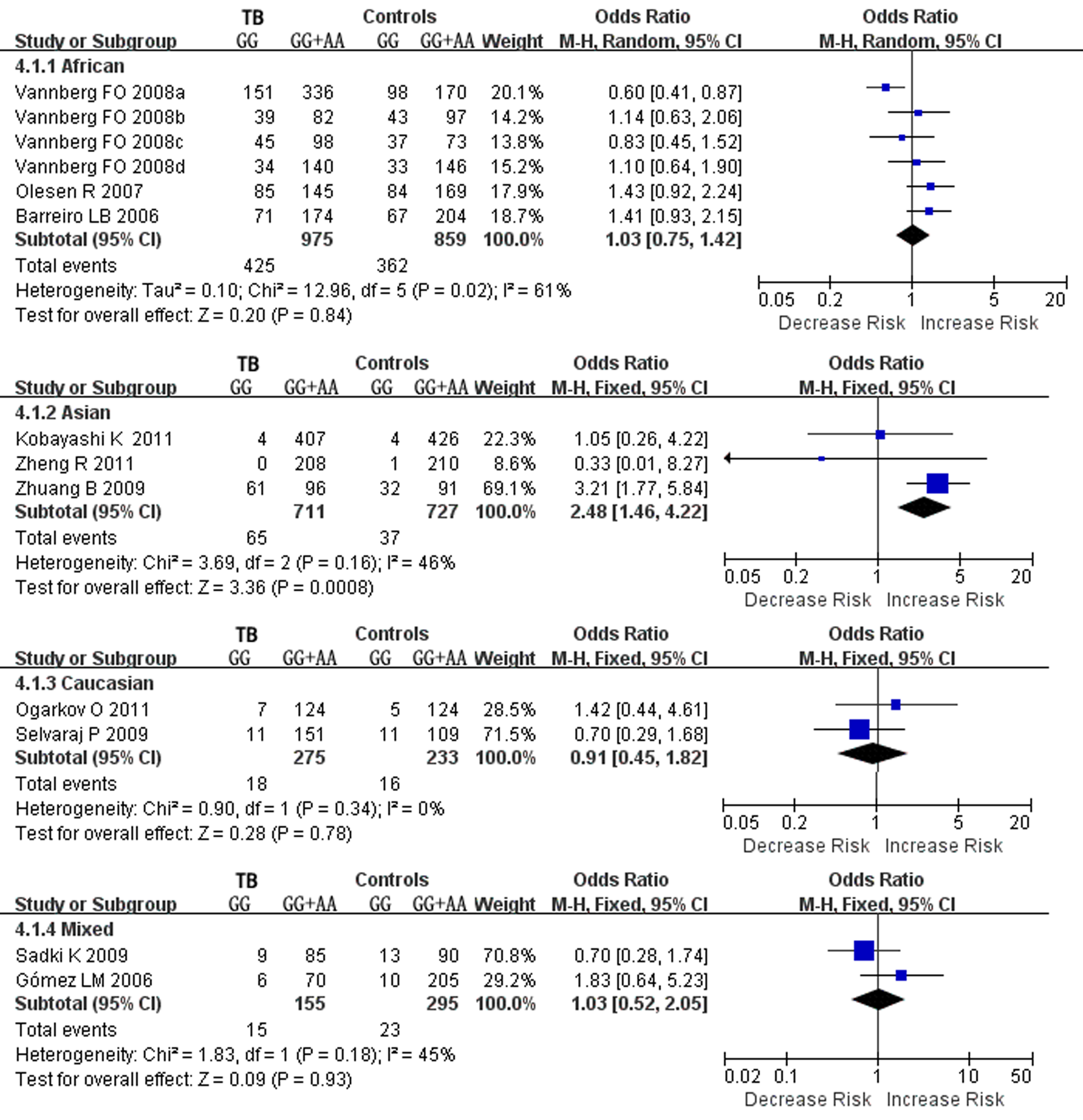

Supplement: Figure S4 — Forest plot of CD209 -336A/G promoter polymorphism and risk of TB in GG vs. AA for each subgroup. The significant association was revealed for Asians in GG vs. AA (OR = 2.48, 95% CI = 1.46–4.22, P = 0.0008). (TIF) [file pone.0041519.s004.tif]

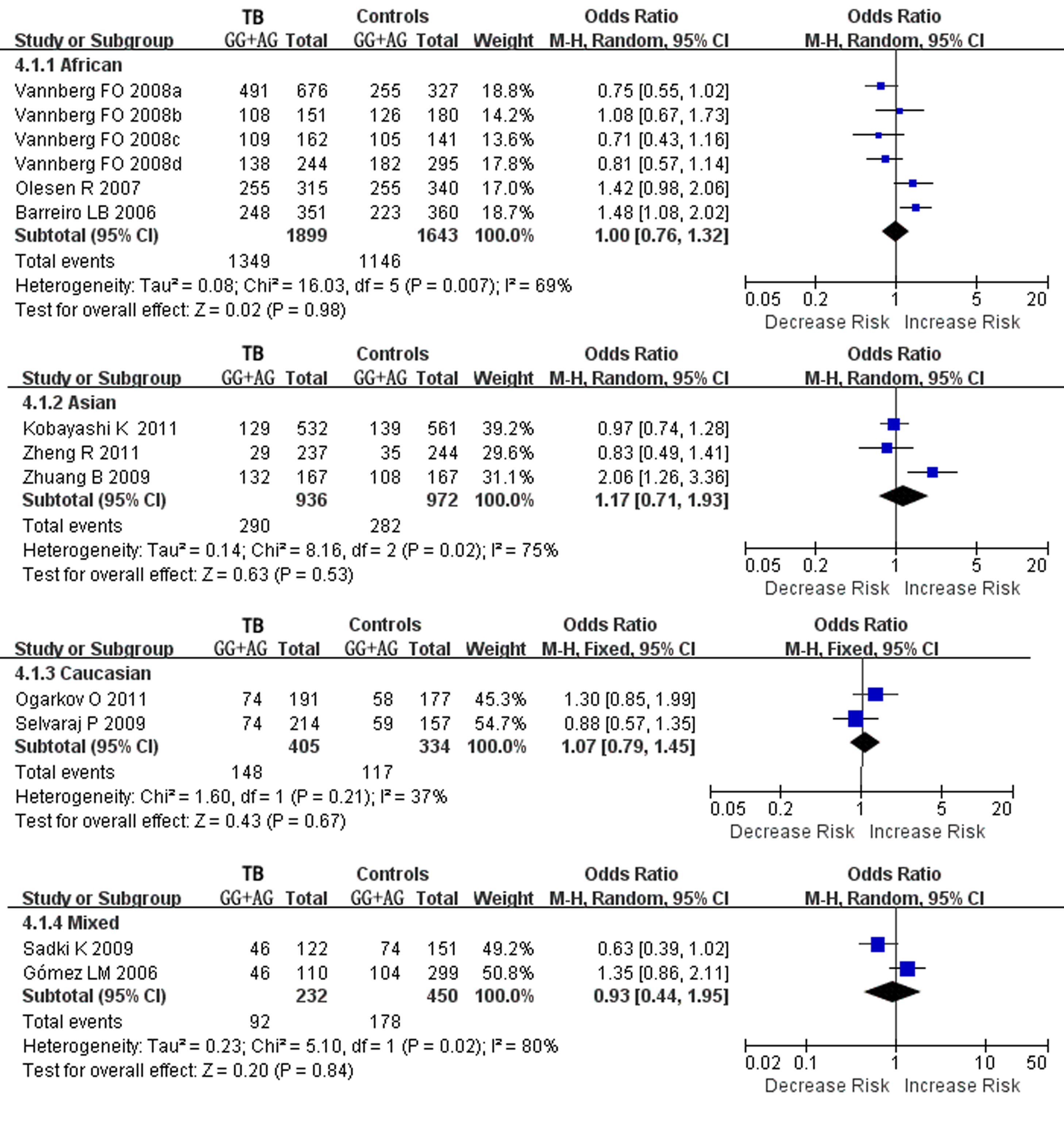

Supplement: Figure S5 — Forest plot of CD209 -336A/G promoter polymorphism and risk of TB in dominant model for each subgroup. No significant association was found between the CD209 -336A/G polymorphism and TB risk in dominant model. (TIF) [file pone.0041519.s005.tif]

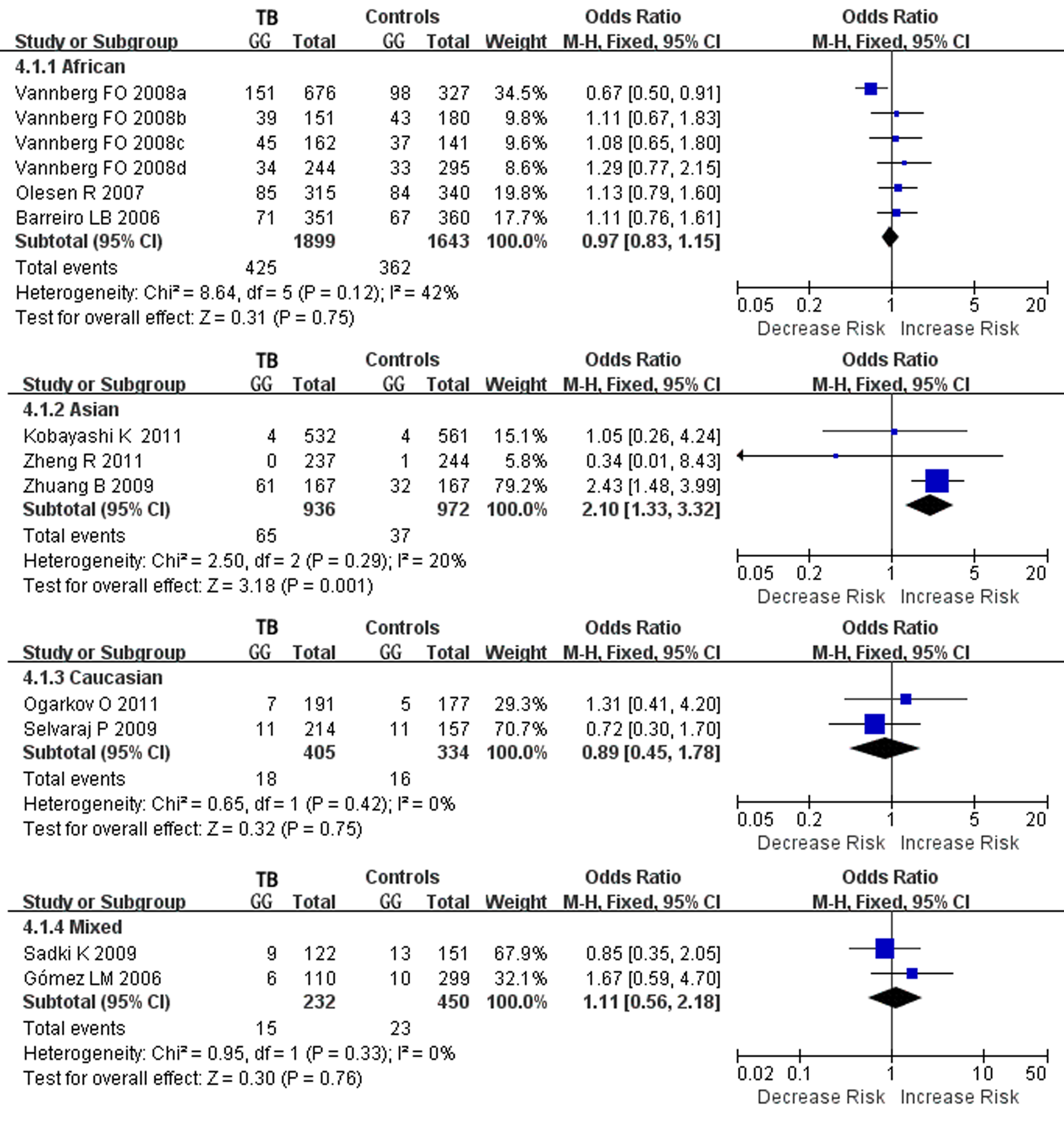

Supplement: Figure S6 — Forest plot of CD209 -336A/G promoter polymorphism and risk of TB in recessive model for each subgroup. The significant association was revealed for Asians in recessive model (OR = 2.10, 95% CI = 1.33–3.32, P = 0.001). (TIF) [file pone.0041519.s006.tif]

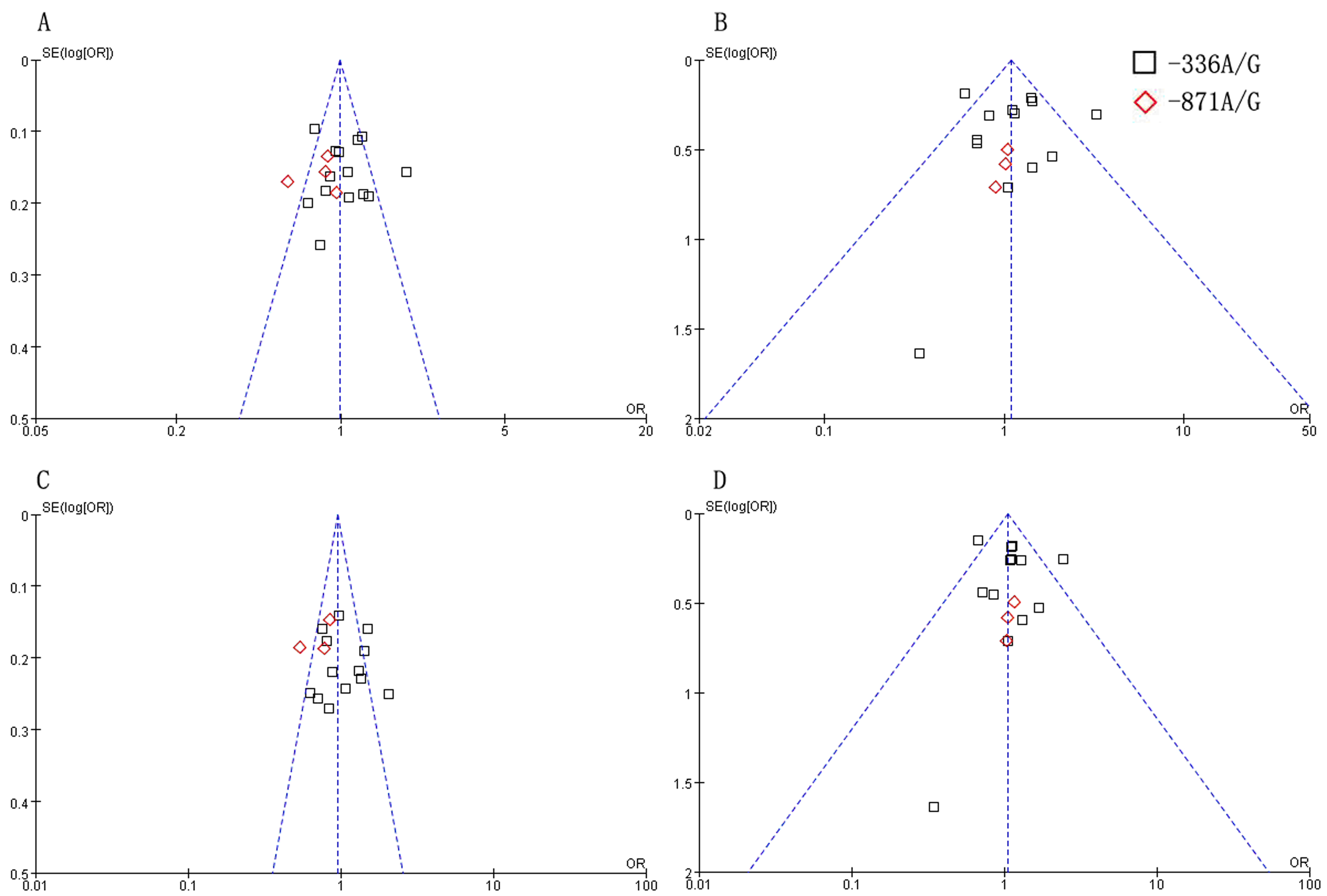

Supplement: Figure S7 — Funnel plots of all genetic models in overall studies. A. G vs. A; B. GG vs. AA; C. dominant model (GG+AG vs. AA); D. recessive model (GG vs. AG+AA). Funnel plots of dominant model seemed asymmetry. Each point represents a separate study for the indicated association. (TIF) [file pone.0041519.s007.tif]

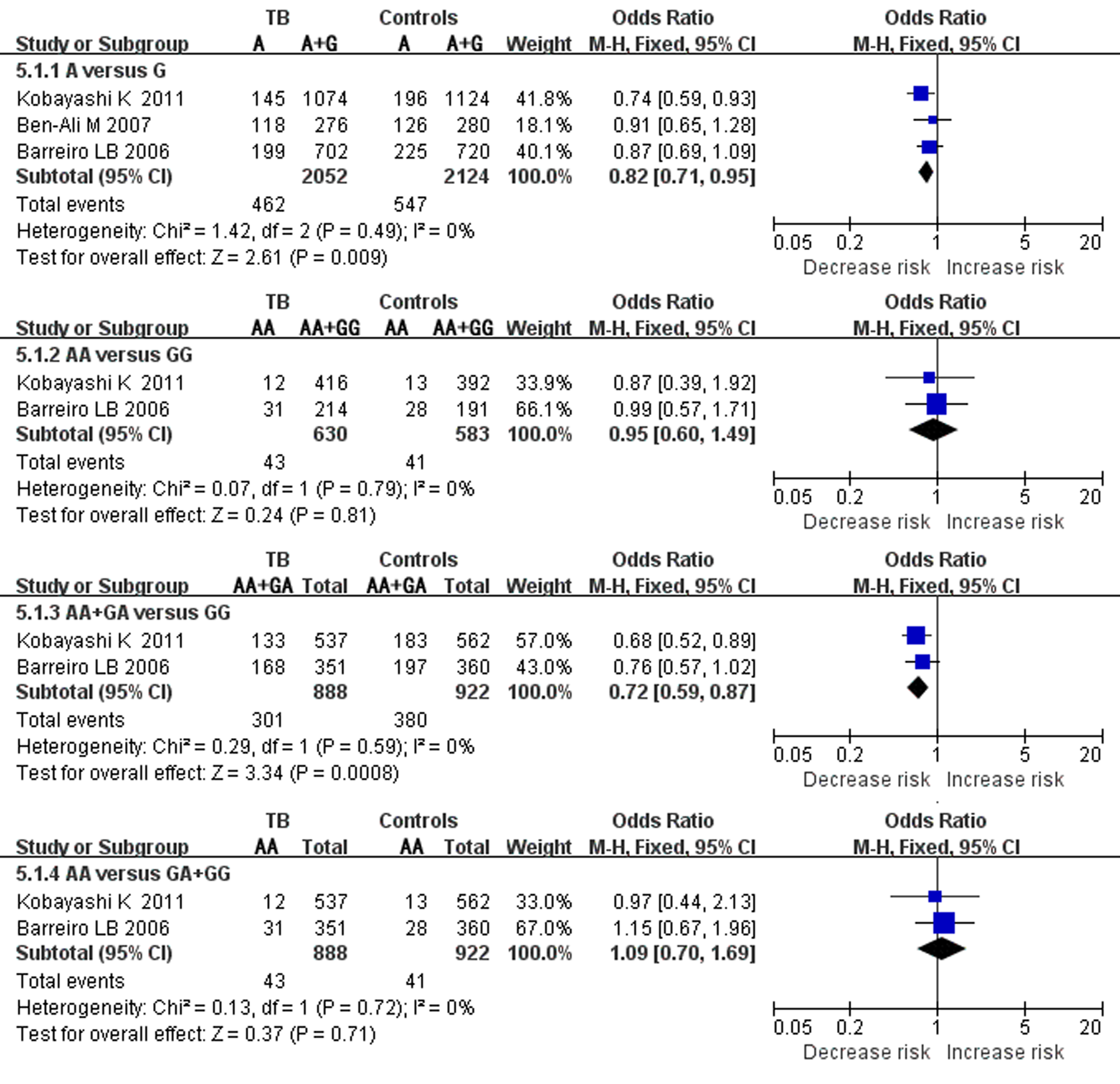

Supplement: Figure S8 — Forest plot of the overall risk of TB associated with the CD209 -939G/A promoter polymorphism. No significant association was found between the CD209 -939G/A polymorphism and TB risk in all genetic models. Error bars indicate 95% CI. Solid squares represent each study in the meta-analysis. Solid diamonds represent pooled OR. (TIF) [file pone.0041519.s008.tif]
