# Supplementary material for: Association between CD209 -336A/G and -871A/G Polymorphisms and Susceptibility of Tuberculosis: A Meta-Analysis
Source: PLoS One. 2012 Jul 24;7(7):e41519. doi: 10.1371/journal.pone.0041519 (PMC3404017; doi:10.1371/journal.pone.0041519)
Supplement: Table S1 — Gene frequencies of all the individual studies used for the meta-analysis. (DOC) [file pone.0041519.s009.doc]

**Table S1 Gene frequencies of all the individual studies used for the meta-analysis**

| Study | Allele 336 cases | | | | |  | Allele 336 controls | | | | |  | Allele 871 cases | | | | |  | Allele 871 controls | | | | |
| --- | --- | --- | --- | --- | --- | --- | --- | --- | --- | --- | --- | --- | --- | --- | --- | --- | --- | --- | --- | --- | --- | --- | --- |
|  | AA | AG | GG | A | G |  | AA | AG | GG | A | G |  | AA | AG | GG | A | G |  | AA | AG | GG | A | G |
| Kobayashi et al. | 403 | 125 | 4 | 931 | 133 |  | 422 | 135 | 4 | 979 | 143 |  | 430 | 102 | 6 | 962 | 114 |  | 435 | 122 | 6 | 992 | 134 |
| Ogarkov et al. | 117 | 67 | 7 | 301 | 81 |  | 119 | 53 | 5 | 291 | 63 |  |  |  |  |  |  |  |  |  |  |  |  |
| Zheng et al. | 208 | 29 | 0 | 445 | 29 |  | 209 | 34 | 1 | 452 | 36 |  | 148 | 80 | 9 | 376 | 98 |  | 138 | 98 | 8 | 374 | 114 |
| Sadki et al. | 76 | 37 | 9 | 189 | 55 |  | 77 | 61 | 13 | 215 | 87 |  |  |  |  |  |  |  |  |  |  |  |  |
| Selvaraj et al. | 140 | 63 | 11 | 343 | 85 |  | 98 | 48 | 11 | 244 | 70 |  |  |  |  |  |  |  |  |  |  |  |  |
| Zhuang et al. | 35 | 71 | 61 | 141 | 193 |  | 59 | 76 | 32 | 194 | 140 |  |  |  |  |  |  |  |  |  |  |  |  |
| Vannberg et al.(a) | 185 | 340 | 151 | 710 | 642 |  | 72 | 157 | 98 | 301 | 353 |  |  |  |  |  |  |  |  |  |  |  |  |
| Vannberg et al.(b) | 43 | 69 | 39 | 155 | 147 |  | 54 | 83 | 43 | 191 | 169 |  |  |  |  |  |  |  |  |  |  |  |  |
| Vannberg et al.(c) | 53 | 64 | 45 | 170 | 154 |  | 36 | 68 | 37 | 140 | 142 |  |  |  |  |  |  |  |  |  |  |  |  |
| Vannberg et al.(d) | 106 | 104 | 34 | 316 | 172 |  | 113 | 149 | 33 | 375 | 215 |  |  |  |  |  |  |  |  |  |  |  |  |
| Ben-Ali et al. | not applicable | | | 200 | 76 |  | not applicable | | | 207 | 73 |  | not applicable | | | 195 | 81 |  | not applicable | | | 195 | 85 |
| Olesen et al. | 60 | 170 | 85 | 290 | 340 |  | 85 | 171 | 84 | 341 | 339 |  |  |  |  |  |  |  |  |  |  |  |  |
| Barreiro et al. | 103 | 177 | 71 | 383 | 319 |  | 137 | 156 | 67 | 430 | 290 |  | 292 | 55 | 4 | 639 | 63 |  | 262 | 94 | 4 | 618 | 102 |
| Gómez et al. | 64 | 40 | 6 | 168 | 52 |  | 195 | 94 | 10 | 484 | 114 |  |  |  |  |  |  |  |  |  |  |  |  |
