# Supplementary material for: Association between CD209 -336A/G and -871A/G Polymorphisms and Susceptibility of Tuberculosis: A Meta-Analysis
Source: PLoS One. 2012 Jul 24;7(7):e41519. doi: 10.1371/journal.pone.0041519 (PMC3404017; doi:10.1371/journal.pone.0041519)
Supplement: Table S3 — Meta-regression analysis of CD209 -336A/G promoter polymorphism and risk of TB after omitting the studies. (DOC) [file pone.0041519.s011.doc]

**Table S3: Meta-regression analysis of *CD209 -336A/G* promoter polymorphism and risk of TB after omitting the studies**

|  | P values | | | |
| --- | --- | --- | --- | --- |
|  | G versus A | GG versus AA | Dominant model | Recessive model |
| Sample size | 0.915 | 0.837 | 0.994 | 0.539 |
| Control source | 0.954 | 0.911 | 0.896 | 0.964 |
| NOS scores | 0.938 | 0.968 | 0.947 | 0.958 |
| frequency of allele | 0.979 | 0.999 | 0.982 | 1.000 |
